# Hematochezia in a patient with liver cirrhosis

**DOI:** 10.1186/1749-7922-2-32

**Published:** 2007-12-04

**Authors:** Ronan A Cahill, Suzanne Norris, Richard B Stephens

**Affiliations:** 1 Department of Surgery, St James's Hospital, Dublin, Ireland; 2 Department of Gastroenterology, St James's Hospital, Dublin, Ireland

## Abstract

Although commonly detectable in patients with cirrhosis, rectal varices only infrequently cause significant hematochezia (0.5–3.6%). While they may be expected to resolve with treatment of the concomitant portal hypertension, there is currently no standardized approach to their management in isolation. Therefore many authorities recommend transjugular intrahepatic portosystemic shunting (TIPS) as a means of alleviating otherwise recalcitrant bleeding. Conceptually, however, rectal varices should be as amenable to local therapies as are their counterparts occurring at the esophagogastric junction. In this report, we describe the use of endoscopic banding per ano to alleviate significant rectal bleeding in a patient with poorly controlled portal hypertension. This allowed medical optimisation so that the underlying pathology could be controlled without recourse to TIPS or other means of creating a formal portosystemic shunt.

## Introduction

Despite the lack of standardised clinical care pathways, it is generally accepted that the treatment of rectal varices focuses on the amelioriation of the underlying pathology- that is the portal hypertension. In cases where the bleeding is especially profuse, the majority consensus seems to be on rapid interventional decompression of the mesenteric circulation either by radiological or surgical means[1-3]. However, in patients whose only symptom is rectal bleeding and whose general medical condition has yet to be physiologically and/or pharmacologically optimized, we suggest that endoscopic intervention may have a role similar to its use in managing patients with bleeding oesophageal varices.

## Case Presentation

**Figure 1:**
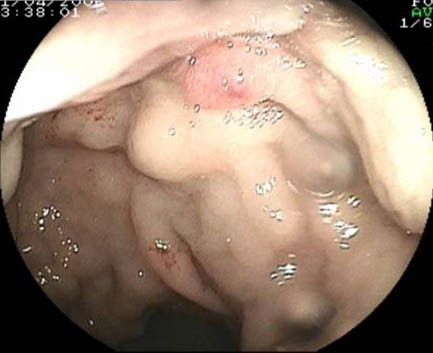
Endoscopic view of the rectum showing rectal varices, one of which demonstrates a red wheal consistent with recent bleeding.

**Figure 2:**
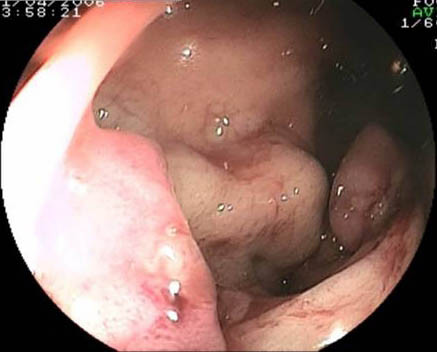
Retroflexed view of rectum again showing the main varix with its bleeding point.

**Figure 3:**
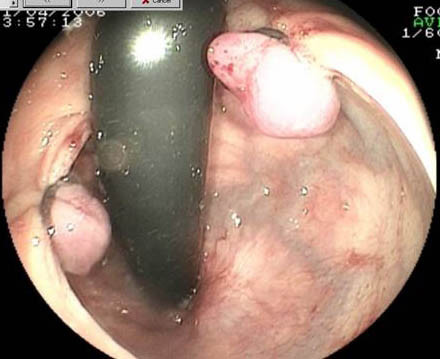
Retroflexed view after endoscopic placement of two bands.

A sixty-eight year old male, known to have Childs B liver cirrhosis secondary to alcohol abuse, presented to our service with persistent passage of bright red blood per rectum. Although hemodynamically normal, the bleeding was associated with a two gram drop in hemoglobin over a 72 hour period and continued despite attempts by medication to reduce his portal venous pressure (he was commenced on propanolol alone for the first 24 hours and then, as the bleeding continued, terlipressin) and correction of his coagulopathy (his INR on admission was 1.7 and so he was administered Vitamin K intravenously). A flexible sigmoidoscopy both confirmed the clinical suspicion of rectal varices (see Figures 1 and 2) and facilitated endoscopic banding of the culprit anomalies (see Figure 3). Significant bleeding ceased immediately after this procedure allowing continued medical optimisation. He was discharged three days later on propanolol (titrated to achieve a resting pulse of 55 bpm), spironolactone and frusemide. Since then he has complied with his medical treatment and the rectal bleeding has not recurred within the first year of follow-up.

## Discussion

Haematochezia requiring acute management is an uncommon problem in patients with portal hypertension (0.5–3.6%) and so treatment paradigms are not well established. Initial medical management following the same principles as that for esophagogastric gastric is generally recommended on both theoretical and empiric grounds and therefore we firstly tried pharmacologically reduction of the patient's portal pressure by instituting both a β-blocker and a synthetic analogue of vasopressin while also addressing his abnormal coagulation. Failure of the bleeding to lessen however mandated an attempt at more definitive intervention. Given that this episode represented the patients first episode of decompensation on a background of Child's B liver disease, we were reluctant to proceed directly to formal mechanical portal vein decompression (by means of portosystemic shunt created either by a Transjugular Intrahepatic approach [i.e. TIPS] or open operation) and therefore considered the available endoscopic treatments proposed in the literature.

Endoscopic band ligation has previously been shown by others to be effective in exactly this scenario albeit in small case series[4,5]as indeed has endoscopic sclerotherapy[6]. Some authors however have expressed concern that the latter procedure risks exacerbation of bleeding when the varices demonstrate red colour signs (as may be seen to be present in image one). While the long-term efficacy of either method is uncertain, such endoscopic intervention has been reported to provide an effective means of short-term relief of rectal bleeding without recourse to formal portal vein decompression in patients with cirrhosis whose main clinical issue is hematochezia. Our own experience encourages us to continue to utilise this therapeutic means in such circumstances.

## References

[B1] O'Connor JB, Issa K (2001). Rectal varices. N Engl J Med.

[B2] Vangeli M, Patch D, Terreni N, Tibballs J, Watkinson A, Davies N, Burroughs AK (2004). Bleeding ectopic varices – treatment with transjugular intrahepatic porto-systemic shunt (TIPS) and embolisation. J Hepatol.

[B3] Nayar M, Saravanan R, Rowlands PC, McWilliams RG, Evans J, Sutton RJ, Gilmore IT, Smart HL, Lombard MG (2006). TIPSS in the treatment of ectopic variceal bleeding. Hepatogastroenterology.

[B4] Firoozi B, Gamagaris Z, Weinshel EH, Bini EJ (2002). Endoscopic band ligation of bleeding rectal varices. Dig Dis Sci.

[B5] Soon MS, Yen HH, Soon A (2005). Endoscopic band ligation for rectal variceal bleeding: serial colonoscopic images. Gastrointest Endosc.

[B6] Wang M, Desigan G, Dunn D (1985). Endoscopic sclerotherapy for bleeding rectal varices: A case report. Am J Gastroenterol.

